# Filamin a binds deleted in liver cancer 1 (DLC1) to promote its tumor suppressor activity and inhibit the SRF coactivator MRTF-A

**DOI:** 10.1016/j.neo.2025.101258

**Published:** 2025-11-28

**Authors:** Michael Sergeev, Melanie A. Meier, Petra Wohlleben, Laura Rupprecht, Mirka Kupraszewicz-Hutzler, Karl Hilgers, Andrea Hartner, Anna-Lena Voegele, Raja Atreya, Yannick Frey, Showmika Srirangan, Jutta Eichler, Caroline Confais, Benoît Hédan, Ulrich Jarry, Monilola A. Olayioye, Susanne Muehlich

**Affiliations:** a Department of Chemistry and Pharmacy, Molecular and Clinical Pharmacy, FAU NeW - Research Center New Bioactive Compounds, Friedrich-Alexander-Universität Erlangen-Nürnberg, D-91058 Erlangen, Germany; b Department of Nephrology and Hypertension, University Hospital of Erlangen, D-91054 Erlangen, Germany; c Department of Pediatrics and Adolescent Medicine, University Hospital of Erlangen, D-91054 Erlangen, Germany; d Department of Medicine 1, University Hospital of Erlangen, D-91054 Erlangen, Germany; e Institute of Cell Biology and Immunology, University of Stuttgart, D-70569 Stuttgart, Germany; f Department of Chemistry and Pharmacy, Medicinal Chemistry, FAU NeW - Research Center New Bioactive Compounds, Friedrich-Alexander-Universität Erlangen-Nürnberg, D-91058 Erlangen, Germany; g Univ Rennes, CNRS, IGDR (Institut de génétique et développement de Rennes) - UMR 6290, F-35000 Rennes, France; h Biotrial Pharmacology, Unité de Pharmacologie Préclinique, Rennes, France; i Univ Rennes, CNRS, INSERM, BIOSIT UAR 3480, US_S 018, Oncotrial, F-35000, Rennes, France

**Keywords:** DLC1, FLNA, MKL1, MRTF, SRF

## Abstract

Filamin A (FLNA) is an actin binding protein that organizes the cytoskeleton and controls many fundamental biological processes, such as cell migration and adhesion. The interaction between FLNA and the Myocardin-related transcription factor A (MRTF-A) promotes the activity of serum response factor (SRF) and cell migration. MRTF-A and SRF play an important role for tumor growth and senescence of hepatocellular carcinoma (HCC). Here, we identified a novel interaction between FLNA and the tumor suppressor Deleted in Liver Cancer 1 (DLC1) *in vitro* and *in vivo* in organoids and mapped the regions of interaction between DLC1 and FLNA. Association with FLNA enhanced DLC1 RhoGAP function, impaired SRF transcriptional activity, and induced cellular senescence. We found a novel molecular switch between the DLC1-FLNA and the MRTF-A-FLNA complexes that is mediated by FLNA phosphorylation at serine 2152. We generated DLC1 binding peptides that dissociate the MRTF-A-FLNA complex and favor the novel DLC1-FLNA complex by preventing actin polymerization and FLNA phosphorylation at serine 2152. Since FLNA phosphorylation at serine 2152 was increased in mouse xenografts, reinforcing the DLC1-FLNA complex by targeting FLNA phosphorylation at serine 2152 represents a promising therapeutic approach for HCC treatment.

## Introduction

Hepatocellular carcinoma (HCC), the most prevalent type of primary liver cancer, is one of the most common solid malignancies worldwide with poor prognosis [1,2]. Globally, it ranks as the sixth most-diagnosed cancer and the third leading cause of cancer-related deaths [3]. Given the large proportion of HCC patients diagnosed at advanced stages and the limited clinical benefit of existing therapies, substantial therapeutic challenges remain. The transcriptional co-activator myocardin-related transcription factor A (MRTF-A) has gained increased attention in HCC formation and liver fibrosis and cirrhosis, precancerous conditions associated with a high probability of developing HCC [4,5]. Targeted therapies have shown encouraging results on suppression of MRTF-A/Serum Response Factor (SRF) transcriptional activity, HCC cell proliferation, and invasion [6].

A key regulator of MRTF-A/SRF signaling is Filamin A (FLNA), which interacts with MRTF-A to promote its transcriptional activity [7]. FLNA is a large actin-binding protein composed of an N-terminal actin-binding domain and 24 immunoglobulin-like (Ig) repeats [8]. Homodimerization via its C-terminal repeat Ig24 enables FLNA to crosslink actin filaments, thus orchestrating cytoskeletal architecture [9]. Complex formation between FLNA and MRTF-A is driven by FLNA phosphorylation at serine 2152 (FLNA pSer^2152^) [10], a post-translational modification which controls association of FLNA with integrins and G protein-coupled receptors (GPCRs) [[11], [12], [13]], enhances cell migration [[14], [15], [16]], and suppresses calpain-mediated cleavage [17,18]. Further research is needed to elucidate the role of FLNA phosphorylation in HCC formation.

The tumor suppressor Deleted in Liver Cancer 1 (DLC1) is a negative regulator of the small GTPase protein RhoA that promotes the hydrolysis of Rho-GTP into Rho-GDP [19,20]. We found that reintroduction of DLC1 induces senescence in HCC cell lines lacking DLC1 expression [21]. In liver tumorigenesis, the induction of senescence acts as a tumor-suppressive mechanism, making DLC1 rescue amenable to HCC therapies [22]. Recent studies indicate that genetic and epigenetic, but also post-translational modifications affect DLC1 expression. DLC1 is a substrate of the cytoplasmic EZH2 (Enhancer of Zeste Homolog 2) methyltransferase, which destabilizes DLC1 through methylation of Lys^678^ to induce its degradation through the ubiquitin-proteasome system [23]. DLC1 proteasomal degradation is governed by CRL4A-FBXW5 ubiquitin ligase complex, while the deubiquitinating enzyme USP7 is involved in the stabilization of DLC1 [24,25]. Phosphorylation of the serine-linker region enhances an intramolecular interaction between the RhoGAP and the DLC1 sterile α motif (SAM) domain, resulting in DLC1 adopting an autoinhibitory closed conformation, which restricts DLC1 functions [26,27]. Tensin-3 (TNS3), specifically its C2 domain, releases DLC1 from autoinhibition through association with the SAM domain, suggesting that DLC1 binding peptides mimicking TNS3-C2 could reactivate DLC1 in HCC [28].

In this study, we identified Filamin A (FLNA) as an interaction partner of DLC1, that enhances its RhoGAP activity and antagonizes MRTF-A/SRF transcriptional activity.

Our findings reveal a functional equilibrium between the tumor-suppressive DLC1-FLNA and oncogenic MRTF-A-FLNA complexes, controlled by FLNA phosphorylation at serine 2152 (FLNA pSer^2152^). Reinforcing the DLC1-FLNA interaction through DLC1 binding peptides or actin depolymerizing agents such as Latrunculin B dissociated the MRTF-A-FLNA interaction, leading to reduced FLNA pSer^2152^ levels, impaired SRF transcriptional activity and the induction of cellular senescence. These findings advance our mechanistic understanding of FLNA regulation by DLC1 and MRTF-A binding, which determines its transcriptional activity, cell migration, differentiation, and cell growth.

## Results

### Identification of FLNA as a DLC1-interacting protein

**Fig. 1:**
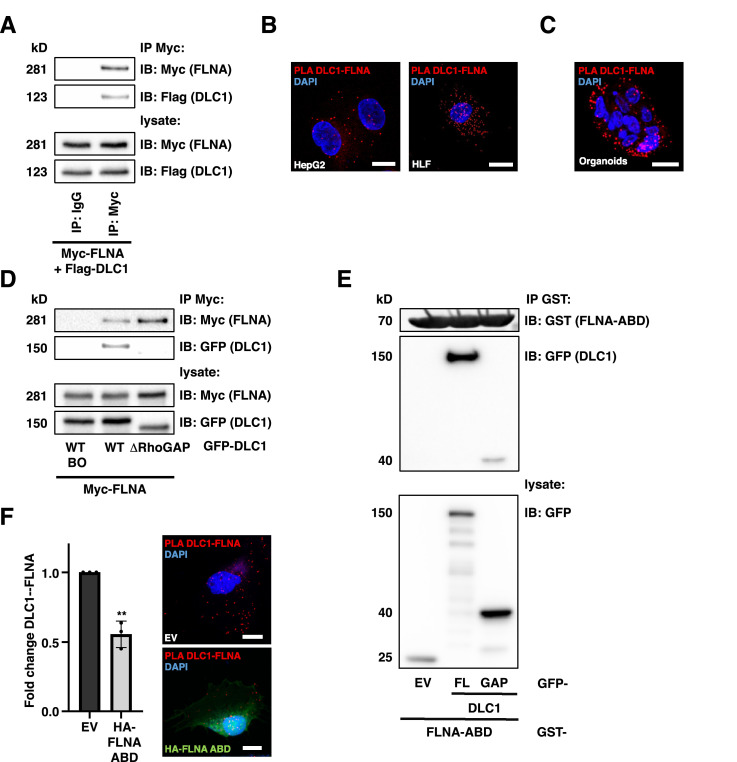
Identification of FLNA as a DLC1-interacting protein. (A) Immunoprecipitation (IP) using anti-Myc antibody in HuH7 cells co-expressing Myc-FLNA and Flag-DLC1, with IgG used as a control. Immunoprecipitates were analyzed by immunoblotting (IB), using anti-DLC1 and anti-FLNA antibodies to detect co-immunoprecipitated Flag-DLC1 and Myc-FLNA. (B) Proximity ligation assays (PLA) for endogenous DLC1 and FLNA in HepG2 and HLF cells. DAPI was used for nuclear staining. Scale bars: 10 µm. Representative images are shown. (C) PLA in human organoids as described in (B). Scale bar: 10 µm. (D) IP and Western blot as in (A) in HuH7 cells expressing Myc-FLNA and either GFP-DLC1 or GFP-DLC1ΔRhoGAP. (E) IP using GST-antibody for GST-FLNA-actin-binding domain (ABD) in HEK293T cells transfected with GFP-empty vector (EV), GFP-DLC1 full length (FL), or the isolated GFP-DLC1 RhoGAP domain (GAP) and immunoblot (IB) with GFP- and GST-antibodies. (F) Immunofluorescence and PLA analysis for endogenous DLC1 and FLNA in HLF cells expressing HA-FLNA-ABD or -empty vector (EV). DAPI was used for nuclear staining. Scale bar: 20µm. Values are mean ± SD (n=3); **p<0.01. Student’s t-test was performed for statistical analysis.

Actin filament cross-linking protein Filamin A (FLNA) is a crucial interaction partner of MRTF-A [7]. Since their complex formation enhanced MRTF-A/SRF signaling, we next searched for FLNA interaction partners that may antagonize MRTF-A/SRF transcriptional activity. In a previous study, mass spectrometry experiments identified FLNA as a putative interactor of the tumor suppressor DLC1 [24]. We first confirmed the interaction by co-immunoprecipitation (Co-IP) assays in HuH7 and HLF HCC cells (Fig. 1A, S1A). To rule out non-specific binding to the beads, a control IgG antibody was used (Fig. 1A). While neither FLNA nor DLC1 was observed in immunoprecipitates with the IgG control antibody (Fig. 1A), DLC1 was recovered from myc- or FLNA immunoprecipitates of HuH7 cells expressing myc-tagged or endogenous FLNA (Fig. 1A, S1A).

Moreover, proximity ligation assays (PLA) verified FLNA as a DLC1 interaction partner in HepG2 and HLF HCC cells, and human organoids (Fig. 1B-C, S1B-C). To map the regions of FLNA critical for its interaction with DLC1, we used a series of HA-tagged constructs spanning the FLNA protein and co-expressed them with Flag-tagged DLC1 in HuH7 cells. Co-IP with Flag antibodies revealed that the N-terminal actin-binding domain of FLNA (FLNA-ABD) [29] is essential for binding to DLC1 (Fig. S1D). Next, we defined segments in DLC1 that are responsible for the interaction with FLNA. Owing to the importance of the RhoGAP domain, we engineered a DLC1 mutant with an internal deletion of a.a. 639-798 (ΔRhoGAP). While wild-type DLC1 (DLC1 WT) associated with FLNA, DLC1ΔRhoGAP abolished FLNA binding in Co-IP assays (Fig. 1D). To ultimately confirm that the FLNA-ABD [29] specifically binds to the DLC1 RhoGAP domain, we generated GST-tagged recombinant proteins. Both the full-length DLC1 protein and the isolated DLC1-GAP domain bound to recombinant GST-FLNA-ABD (Fig. 1E). Given the interaction between DLC1 and the FLNA actin-binding domain (FLNA-ABD), we next investigated whether expression of HA-tagged FLNA-ABD could interfere with the DLC1-FLNA complex by competing with endogenous FLNA for DLC1 binding. PLA assays confirmed the dissociation of the DLC1-FLNA complex upon ectopic expression of the FLNA-ABD domain (Fig. 1F). These data reveal an interaction between DLC1 and FLNA via the DLC1 RhoGAP domain and the FLNA actin-binding domain.

### Interaction with FLNA promotes DLC1 RhoGAP activity and induces senescence

**Fig. 2:**
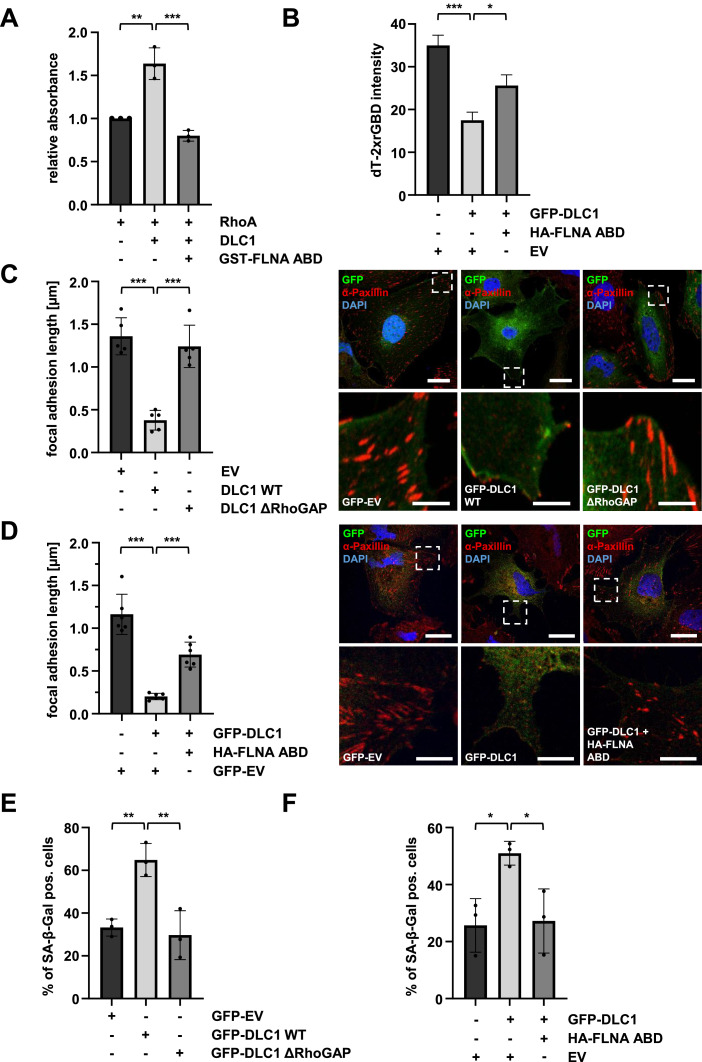
**Interaction with FLNA promotes DLC1 RhoGAP activity and induces senescence.** (A) RhoGAP activity was measured by assessing RhoA-GTP hydrolysis, indicated by CytoPhos absorbance at 650 nm. Each reaction contained reaction buffer, GTP, RhoA, and DLC1, with or without GST-FLNA-actin-binding domain (ABD). Values are mean ± SD (n=3); **p<0.01, ***p<0.001. One-way ANOVA followed by Tukey’s post-hoc test was used for statistical analysis. (B) Quantitation of fluorescence intensity in HeLa cells stably expressing the Rho biosensor dT-2xrGBD under the control of a tetracycline response element (TRE). HeLa cells were transfected with GFP-DLC1 and HA-FLNA ABD or empty vector (EV), followed by treatment with 1 µg/ml doxycycline for 18 hours to induce dT-2xrGBD expression. Data represent mean fluorescence intensity per cell ± SEM (n=40). *p<0.05, ***p<0.001. Representative images are shown in Fig. S2B. (C) Quantification of focal adhesion length, determined by immunostaining with an anti-Paxillin antibody in HuH7 cells expressing GFP DLC1 WT, GFP DLC1ΔRhoGAP, or GFP-empty vector (EV). DAPI was used for nuclear staining. Scale bars: 20 µm (upper panel) and 5 µm (lower panel). Values are mean ± SD (n=5 fields of view); ***p<0.001. (D) Quantification of focal adhesion length, determined by Paxillin immunostaining in HuH7 cells expressing GFP DLC1 WT together with HA-FLNA ABD or –empty vector (EV). DAPI was used for nuclear staining. Scale bars: 20 µm (upper panel) and 5 µm (down panel). Values are mean ± SD (n=6 fields of view); ***p<0.001. (E) Quantification of senescence-associated β-galactosidase (SA-β-gal) staining in HuH7 cells treated as in (C). Cells were fixed 5 days post-transfection, and the number of SA-β-gal-positive blue cells was counted. Values are mean ± SD (n=3); *p<0.05, **p<0.01. (F) Quantification of SA-β-gal staining of HuH7 cells, expressing the same constructs as (D). Cells were fixed 5 days post-transfection, and the number of SA-β-gal-positive blue cells was counted. Values are mean ± SD (n=3); *p<0.05. One-way ANOVA followed by Tukey’s post-hoc test was used for statistical analysis.

Given that FLNA and DLC1 associate via the DLC1 RhoGAP domain, we next examined the effect of FLNA ABD binding on DLC1’s GAP activity by measuring GTP hydrolysis. As expected, the GAP domain of DLC1 significantly increased GTP hydrolysis, which was prevented by FLNA-ABD (Fig. 2A). By using a Rho biosensor dT-2xrGBD containing two rhotekin domains that bind active, GTP-bound RhoA and a dimericTomato domain for fluorescence imaging (dT-2xrGBD), we were able to visualize endogenous RhoA activity [30]. As anticipated, fluorescence intensity decreased upon DLC1 transfection, which was reversed by ectopic expression of FLNA-ABD (Figs. 2B, S2A), indicating that association with FLNA is critical for DLC1 RhoGAP activity and the RhoA activation status.

To further corroborate that interaction with FLNA impairs established DLC1-dependent cellular responses, we assessed focal adhesion length, changes in cytoskeletal dynamics, and senescence induction [21,[31], [32], [33]]. Transfection with DLC1 WT, but not the DLC1 ΔRhoGAP mutant markedly reduced focal adhesion length and stress fiber formation in HuH7 cells (Figs. 2C, S2B). The DLC1-mediated reduction in focal adhesion length and stress fiber disassembly was prevented upon ectopic expression of the FLNA ABD (Figs. 2D, S2C-D). Given our finding that DLC1 rescue drives senescence induction [21], we determined senescence-associated ß-galactosidase (SA-β-gal) activity upon DLC1 expression in HuH7 cells [21]. As expected, DLC1 WT significantly increased SA-β-gal activity, while the ΔRhoGAP mutant showed no effect (Fig. 2E). Similarly, co-expression of the FLNA-ABD counteracted the DLC1-dependent senescence induction (Fig. 2F). These results demonstrate that association with FLNA promotes DLC1 RhoGAP activity and induces cellular senescence.

### DLC1 activation through DLC1 binding peptides promotes FLNA-DLC1 association and cellular senescence

**Fig. 3:**
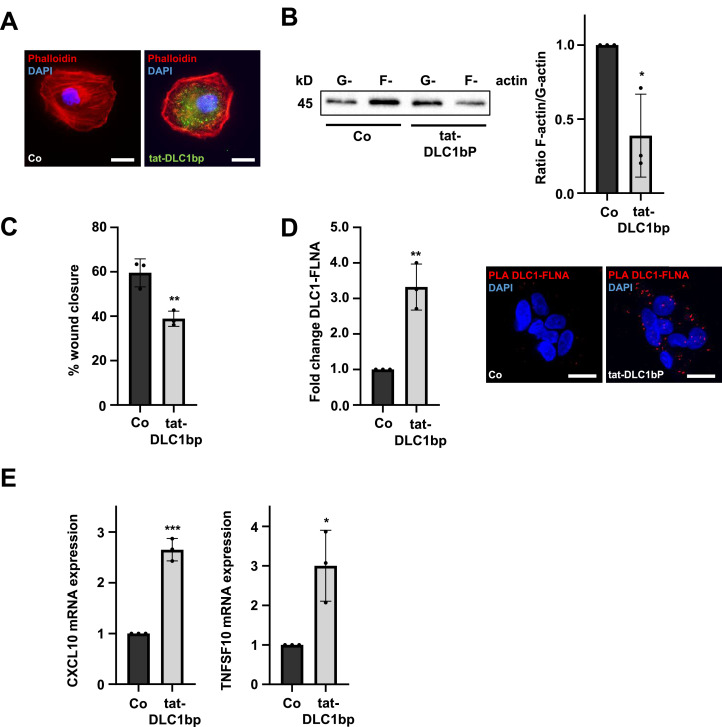
**DLC1 activation through DLC1 binding peptides promotes DLC1-FLNA association and cellular senescence.** (A) Immunofluorescence analysis in HuH7 cells stained with Alexa Fluor 555 phalloidin upon treatment with 20 µM tat-DLC1 binding peptide (tat-DLC1bp) or DMSO (Co) for 1 h. DAPI was used for nuclear staining. Scale bar: 20 µm. (B) Immunoblot analysis of lysates from HuH7 cells treated as in (A) using anti-actin antibody (left). Quantification of the F-/G-actin ratio (right); Values are mean ± SD (n=3); *p<0.05. (C) Quantification of scratch-wound assay in HuH7 cells treated as in (A). Images were taken 48 h post-treatment and analyzed using ImageJ. Representative images are shown in Fig. S3B. Values are mean ± SD (n=3); **p<0.01. (D) Quantification of proximity ligation assay (PLA) for endogenous DLC1 and FLNA and representative images of human organoids treated with 20 µM tat-DLC1bp or DMSO (Co) for 4 h (left). DAPI was used for nuclear staining. Scale bar: 10 µm. Values are mean ± SD (n=3); **p<0.01. (E) HuH7 cells treated with 30 μM tat-DLC1bp or DMSO (Co) every 48 h for 5 days were subjected to quantitative real-time PCR (qRT-PCR). CXCL10 and TNFSF10 expression levels were measured and normalized to 18S rRNA. Values are mean ± SD (n = 3); *p<0.05, ***p < 0.001. Student’s t-test was performed for statistical analysis.

To further elucidate the molecular mechanism underlying the complex formation between DLC1 and FLNA, we explored a therapeutic approach targeting endogenous DLC1. For this approach, we generated a DLC1 binding peptide (DLC1bp) comprising residues 244-255 of the C2 domain of tensin-3 (TNS3-C2), which activates DLC1 by interfering with an intramolecular interaction between the RhoGAP and SAM domains [28]. To facilitate cellular uptake, the peptide was also fused to a cell-penetrating peptide whose sequence was derived from the HIV-1 tat protein [34], resulting in fusion peptide tat-DLC1bp (see Supplementary Table S10 for peptide sequences). First, we validated the efficacy of tat-DLC1bp in HCC cells by phalloidin staining to confirm the disassembly of the actin cytoskeleton [32]. Upon successful cellular uptake, tat-DLC1bp treatment led to a marked depolymerization of stress fibers in HuH7 and Hep3B cells (Figs. 3A, S3A) and significantly shifted the F-/G-actin ratio toward monomeric G-actin (Figs. 3B), consistent with DLC1 activation and downstream RhoA inactivation. Since changes in cytoskeletal dynamics affect cell migration [35,36], we next performed scratch wound assays to determine whether tat-DLC1bp-induced DLC1 activation impairs cell migration. Indeed, wound closure in HuH7 and HuH6 cells was significantly reduced following tat-DLC1bp treatment (Figs. 3C, S3B-C).

Importantly, PLA assays in human organoids and HuH7 cells confirmed that tat-DLC1bp induced DLC1 activation treatment promoted DLC1-FLNA complex formation *in vitro* and *in vivo* (Figs. 3D, S3D). Similar to ectopically expressed DLC1, tat-DLC1bp induced complex formation between DLC1 and FLNA led to the induction of cellular senescence, as revealed by SA-β-gal staining and increased mRNA levels of markers of the senescence-associated secretory phenotype (SASP) CXCL10 and TNFSF10 [[37], [38], [39], [40]] (Figs. 3E, S3E). To rule out nonspecific effects mediated by the cell penetrating tat-sequence, a control peptide consisting solely of the tat domain (tat-only) was used. This control treatment did not induce cellular senescence, as did the tat-DLC1 bp (Fig. S3E).

In summary, these findings provide evidence that tat-DLC1bp treatment effectively promotes the DLC1-FLNA interaction, leading to the induction of cellular senescence.

### DLC1-FLNA complex formation inhibits MRTF-A/SRF transcriptional activity

**Fig. 4:**
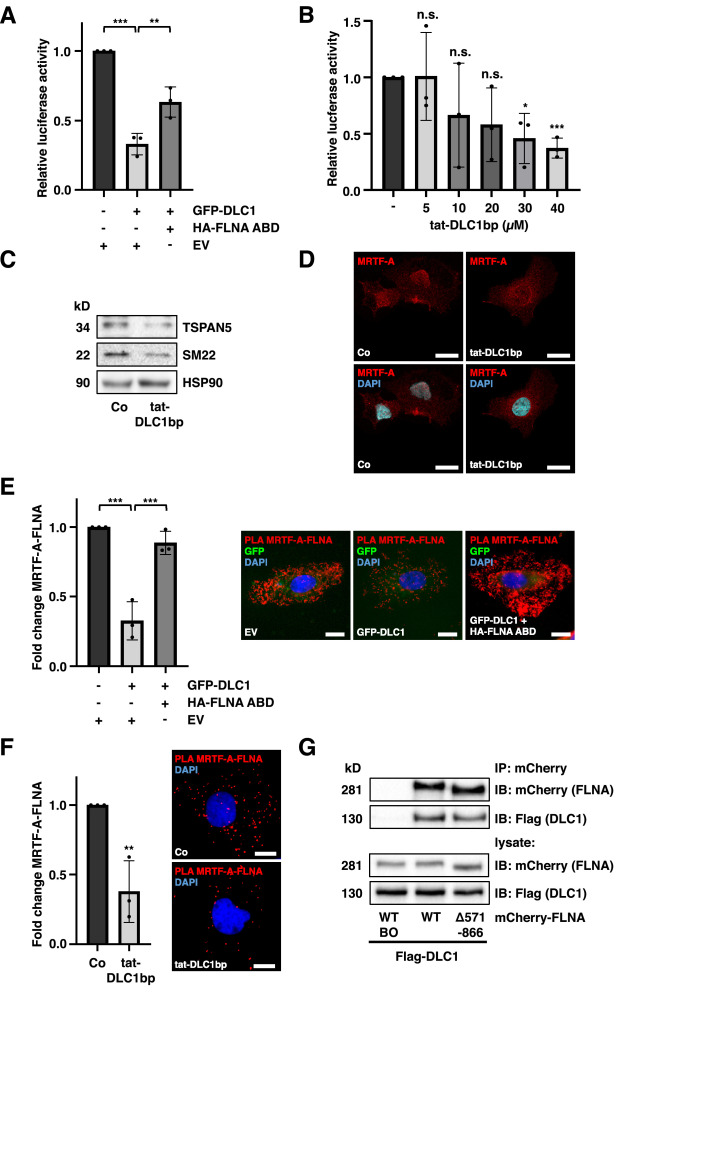
**DLC1-FLNA complex formation inhibits MRTF-A/SRF transcriptional activity.** (A) Luciferase activity in HuH7 cells co-transfected with an SRE-dependent firefly luciferase reporter gene (5xSRE), a Renilla luciferase internal control (pRL-SV40P), GFP-DLC1, and HA-FLNA 2-275 (ABD) or -empty vector (EV). Firefly luciferase activity was measured and normalized to Renilla luciferase activity. Values are mean ± SD (n = 3); *p<0.05, ***p<0.001; One-way ANOVA followed by Tukey’s post-hoc test was used for statistical analysis. (B) HuH7 cells expressing 5xSRE and pRL-SV40P were treated with tat-DLC1 binding peptide (tat-DLC1bp) using the indicated concentrations. After 24 h, Firefly luciferase activity was measured and normalized to Renilla luciferase. Values are mean ± SD (n = 3); *p<0.05, ***p<0.001; Student’s t-test was performed for statistical analysis. (C) Immunoblot for SM22 and TSPAN5 in HuH7 cells treated with 30 µM tat-DLC1bp or DMSO (Co) for 24 h. (D) Immunofluorescence analysis of MRTF-A in HuH7 cells treated as in (C). DAPI was used for nuclear staining. Scale bar: 20 µm. (E) Quantification of proximity ligation assay (PLA) signals for MRTF-A and FLNA in HuH7 cells expressing GFP-DLC1 together with HA-FLNA ABD or –empty vector (EV). DAPI was used for nuclear staining. Scale bar: 20 µm. Values are mean ± SD (n=3); **p<0.01; One-way ANOVA followed by Tukey’s post-hoc test was used for statistical analysis. (F) PLA analysis for MRTF-A-FLNA interactions in HuH7 cells treated with 30 µM tat-DLC1bp or DMSO (Co) for 4 h. Values are mean ± SD (n=3); **p<0.01; One-way ANOVA followed by Tukey’s post-hoc test was used for statistical analysis. (G) Lysates from HuH7 cells co-expressing Flag-DLC1 with either mCherry-FLNA WT or mCherry-FLNA Δ571-866 were subjected to immunoprecipitation (IP) using an anti-mCherry antibody. Immunoblot analysis (IB) was performed with anti-mCherry and anti-Flag antibodies. BO: Dynabeads-only control (no antibody).

To delineate the impact of the DLC1-FLNA interaction on MRTF-A/SRF transcriptional activity, we performed reporter gene assays with a 5xSRE luciferase reporter gene in HuH7 cells transfected with GFP-DLC1 and the FLNA-ABD. We obtained a strong reduction of luciferase activity in GFP-DLC1 expressing cells. In contrast, the FLNA-ABD prevented the suppression of SRF reporter gene activity upon GFP-DLC1 expression in HuH7 cells (Fig. 4A). Next, we evaluated the efficacy of the DLC1 binding peptide (tat-DLC1bp) in inhibiting SRF activity. Titration experiments with the tat-DLC1bp revealed a dose-dependent reduction in SRF activity in HuH7 and HuH6 cells (Figs. 4B, S4A). Consistent with this, MRTF-A nuclear localization and expression of MRTF/SRF-dependent target genes TSPAN5 and SM22 [41,42] were also reduced upon administration of the DLC1bp, as determined by immunoblotting and immunofluorescence analysis (Figs. 4C-D, S4B-C).

According to our previous studies, the interaction between FLNA and MRTF-A facilitates MRTF-A/SRF target gene expression [7]. We therefore performed PLA assays to analyze DLC1’s impact on the MRTF-FLNA complex. DLC1 expression significantly reduced MRTF-A-FLNA PLA signals, which was prevented by co-expression of the FLNA-ABD (Fig. 4E). Likewise, tat-DLC1bp treatment reduced MRTF-A-FLNA interactions in the PLA assay (Fig. 4F). In order to determine whether this MRTF-A-FLNA complex dissociation is mediated via DLC1 as a negative regulator of RhoA activity, we co-expressed constitutively active RhoA (RhoAV14) with DLC1. RhoAV14 restored the MRTF-A-FLNA interaction in the presence of DLC1 (Fig. S4D). We next tested whether MRTF-A binding is a prerequisite for DLC1 association with FLNA by using a FLNA mutant (mCherry-FLNA Δ571-866) [7] incapable of binding MRTF-A. Immunoprecipitation with anti-mCherry antibody revealed that a robust DLC1 interaction could be observed for the FLNA mutant devoid of the MRTF-A binding domain (Fig. 4G).

These results underscore the crucial role of the DLC1-FLNA interaction for SRF transcriptional activity, MRTF-FLNA complex formation, and MRTF-dependent target gene expression.

### FLNA phosphorylation governs the equilibrium between DLC1-FLNA and MRTF-A-FLNA complexes

**Fig. 5:**
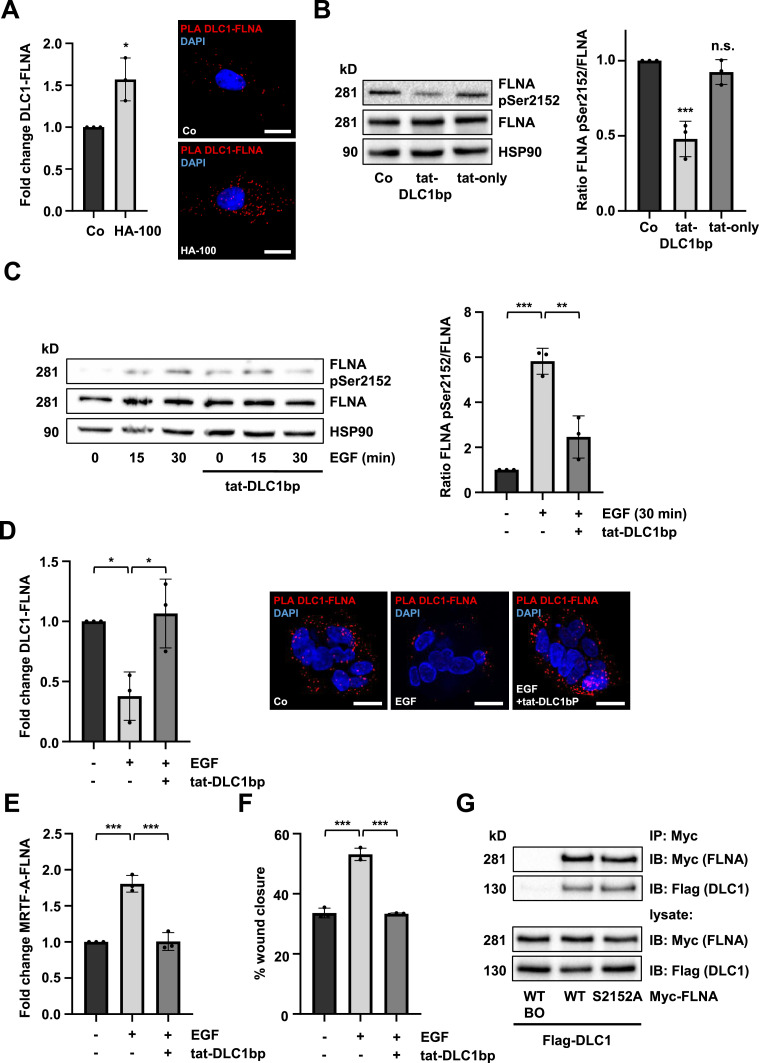
**FLNA phosphorylation governs the equilibrium between the DLC1-FLNA and MRTF-A-FLNA complexes.** (A) Quantification of proximity ligation assay (PLA) signals for endogenous DLC1-FLNA interactions in HuH7 cells treated with 10 µM HA-100 or DMSO (Co). DAPI was used for nuclear staining. Scale bar: 20 µm. Values are mean ± SD (n=3); *p<0.05. (B) Immunoblot for FLNA pSer^2152^, total FLNA, and HSP90 in HuH7 cells treated with 30 µM tat-DLC1-binding peptide (tat-DLC1bp), tat-control peptide (tat-only), or DMSO (Co) for 4 h. The FLNApSer^2152^/FLNA ratio was quantified and normalized to HSP90. Values are mean ± SD (n=3); **p<0.01. (C) Serum-starved Hep3B cells (0.2% FBS, 16 h) were preincubated with 30 µM tat-DLC1bp or DMSO (Co) for 45 min, followed by stimulation with 100 ng/ml EGF for the indicated time points. Immunoblot analysis was performed for FLNA pSer^2152^, total FLNA, and HSP90. The FLNApSer^2152^/FLNA ratio was quantified and normalized to HSP90. Values are mean ± SD (n=3); **p<0.01, ***p<0.001. (D) Quantification of proximity ligation assay (PLA) signals for endogenous DLC1-FLNA interactions in serum-starved human organoids preincubated with 30 µM tat-DLC1bp or DMSO (Co) for 45 min, followed by stimulation with 100 ng/ml EGF for 30 min. DAPI was used for nuclear staining. Scale bar: 10 µm. Values are mean ± SD (n=3); *p<0.05 **p<0.01. (E) PLA analysis for endogenous MRTF-A and FLNA in serum-starved Hep3B cells (0.2% FBS for 16h), treated as described in (C). Values are mean ± SD (n=3); **p<0.01, ***p<0.001. (F) Quantification of wound closure in Hep3B cells subjected to scratch-wound assay following preincubation with tat-DLC1bp and treatment with 100 ng/ml EGF. Images were taken 24 h post-scratch and analyzed using ImageJ. Values are mean ± SD (n=3); ***p<0.001. (G) Immunoprecipitation (IP) for Myc-FLNA in HuH7 cells expressing Flag-DLC1 and either Myc-FLNA WT or Myc-FLNA S2152A. Immunoblot (IB) analysis was performed using anti-Myc- and anti-Flag antibodies. BO: Dynabeads-only control (no antibody). One-way ANOVA followed by Tukey’s post-hoc test was used for statistical analysis.

The association of FLNA and DLC1 in the absence of MRTF-A prompted us to hypothesize that there is an equilibrium between the two complexes. Our group recently identified FLNA phosphorylation at serine 2152 as the key molecular mechanism driving its association with MRTF-A [10]. To determine whether the FLNA phosphorylation status affects its binding to DLC1, we used the protein kinase inhibitor HA-100, which abrogated MRTF-A-FLNA complex formation [10]. HA-100 treatment reduced FLNA pS2152 levels, as determined by immunoblotting using an antibody raised against FLNA pSer^2152^ (Fig. S5A). PLA analyses revealed increased DLC1-FLNA complex formation upon administration of HA-100 (Fig. 5A). We next assessed the FLNA phosphorylation status upon DLC1bp treatment and observed a significant reduction of FLNA pSer^2152^ in HuH7 and HuH6 cells following tat-DLC1bp treatment (Fig. 5B, S5B). As expected, the tat-control peptide (tat-only) did not affect FLNA pSer^2152^ levels (Fig. 5B).

We chose epidermal growth factor (EGF) as a well-established stimulus for FLNA phosphorylation at S2152 [14] to assess whether EGF may shift the equilibrium towards the MRTF-A-FLNA complex. Stimulation with EGF increased FLNA pSer^2152^ levels in serum-starved Hep3B cells, which was prevented by the tat-DLC1bp (Fig. 5C). PLA assays in Hep3B cells and organoids confirmed that DLC1 dissociates from FLNA upon EGF treatment, whereas preincubation with tat-DLC1bp reinforced the DLC1-FLNA complex (Figs. 5D, S5C). In contrast, analysis of the MRTF-A-FLNA interaction showed inverse results. (Figs. 5E, S5D). Since dissociation of the MRTF-A-FLNA complex suppresses cell motility [7], we assessed EGF treatment with or without preincubation of tat-DLC1bp. As expected, tat-DLC1bp inhibited the EGF-induced cell migration (Fig. 5F).

To corroborate the latter findings, we used lysophosphatidic acid (LPA) as an alternative stimulus to induce MRTF-A binding to FLNA [10]. Both PLA and FRET assays demonstrated that LPA treatment similarly promoted DLC1-FLNA complex dissociation (Fig. S5E). Co-IP analysis using a non-phosphorylatable FLNA S2152A [43] mutant revealed increased DLC1 binding (Fig. 5G), ultimately confirming FLNA phosphorylation as the underlying mechanism driving its dissociation from DLC1 in favor of MRTF-A binding.

### Actin dynamics modulate the FLNA phosphorylation status and association with DLC1 or MRTF-A

**Fig. 6:**
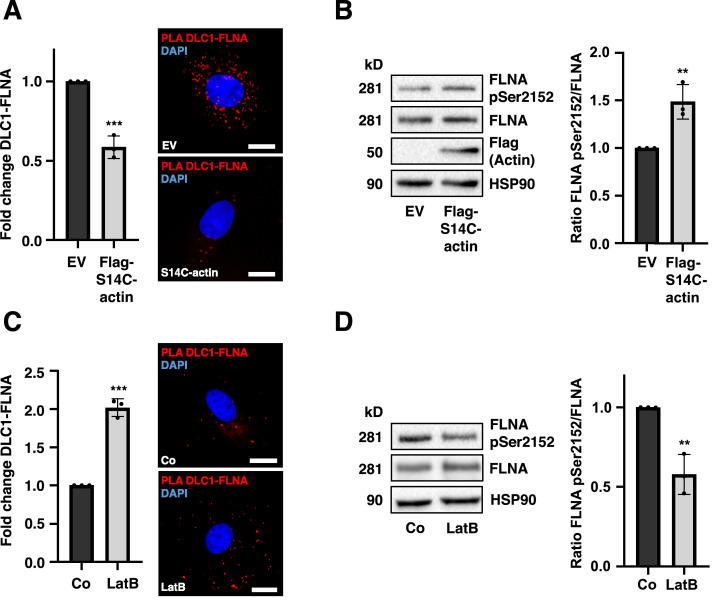
**Actin dynamics modulate the FLNA phosphorylation status and association with DLC1 or MRTF-A.** (A) Quantification of proximity ligation assay (PLA) for DLC1-FLNA interactions in HepG2 cells expressing pcDNA3.1-empty vector (EV) or Flag-S14C-actin. DAPI was used for nuclear staining. Scale bar: 20 µm. Values are mean ± SD (n=3); ***p<0.001. (B) HepG2 cells have been treated as in (A). 16 h post-starvation, cells were lysed and immunoblotted using the indicated antibodies (left). Quantification of the FLNApSer^2152^/FLNA ratio, normalized to HSP90 (right). Values are mean ± SD (n=3); **p<0.01. (C) PLA analysis for DLC1-FLNA interactions in HuH7 cells treated with 1 µM Latrunculin B (LatB) or DMSO (Co) for 24 h. DAPI was used for nuclear staining. Scale bar: 20 µm. Values are mean ± SD (n=3); ***p<0.001. (D) Immunoblot analysis of HuH7 cell lysates treated with 1 µM Latrunculin B (LatB) or DMSO (Co) for 24 h using the indicated antibodies (left). Quantification of the FLNApSer^2152^/FLNA ratio, normalized to HSP90 (right). Values are mean ± SD (n=3); *p<0.05, **p<0.01. Student’s t-test was performed for statistical analysis.

**Fig. 7:**
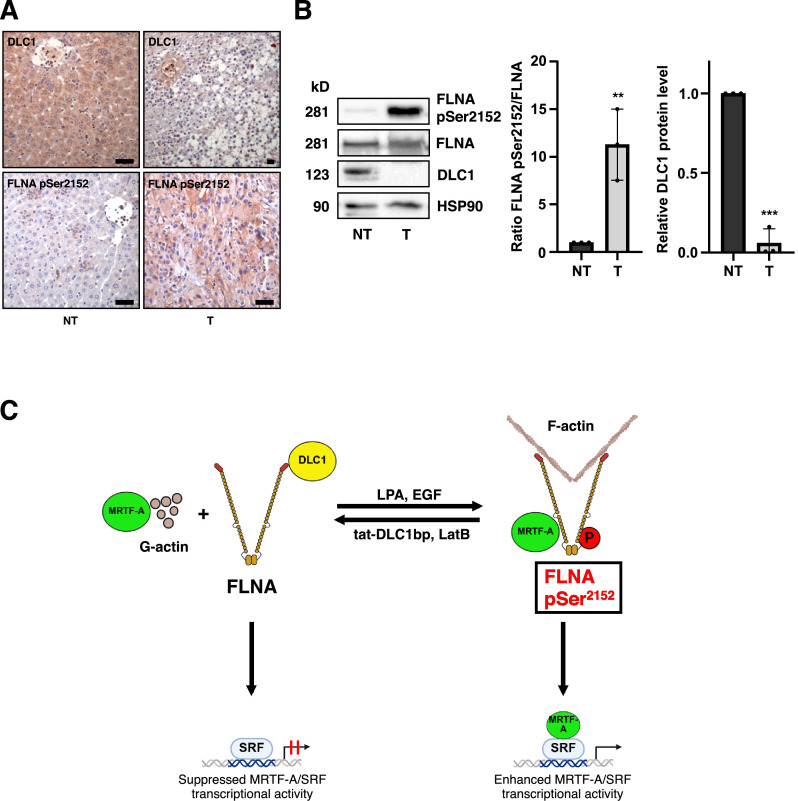
**Enhanced FLNA phosphorylation at S2152 in tumor xenografts.** (A) Immunohistochemical staining of formalin-fixed, paraffin-embedded sections from mouse tumor xenografts (T) and non-tumorous liver samples (NT) for DLC1 and FLNA pSer^2152^. Scale bar: 50 µm. (B) Immunoblot analysis of lysates from mouse tumor xenografts (T) and non-tumorous liver samples (NT), using the indicated antibodies. The FLNApSer^2152^/FLNA ratio and DLC1 expression were quantified and normalized to HSP90. Values are mean ± SD (n=3); **p<0.01, ***p<0.001. Student’s t-test was performed for statistical analysis. (C) Schematic model for the regulation of FLNA by interaction with DLC1 and MRTF-A. FLNA interacts with DLC1, thereby enhancing its RhoGAP activity. FLNA phosphorylation at S2152 upon EGF or LPA stimulation switches FLNA-binding to MRTF-A, resulting in enhanced MRTF/SRF transcriptional activity. In contrast, impairment of FLNA phosphorylation at S2152 by the actin polymerization inhibitor Latrunculin B or the DLC1-binding peptide promotes DLC1-FLNA complex formation, which inhibits MRTF-A/SRF transcriptional activity.

The essential role of FLNA as an actin binding protein for association with MRTF-A prompted us to test the hypothesis that the cellular F-/G-actin content status may affect the association of FLNA and DLC1. Indeed, polymerized F-actin, encoded by the plasmid S14C-actin [44], impaired DLC1-FLNA complex formation in PLA assays (Fig. 6A). This reduction in DLC1-FLNA interactions correlated with elevated FLNA pSer^2152^ levels, as shown by immunoblotting (Fig. 6B). Furthermore, administration of Latrunculin B (LatB), a potent inhibitor of actin depolymerization [45], significantly increased the association between DLC1 and FLNA, as assessed by PLA (Fig. 6C). LatB treatment reduced FLNA pSer^2152^ levels (Fig. 6D), consistent with our prior findings that non-phosphorylated FLNA has a higher binding affinity for DLC1 (Fig. 5G). These data suggest a model depicted in Fig. 7C in which elevated cellular F-actin content and enhanced FLNA phosphorylation at S2152 lead to increased MRTF-A-FLNA association, whereas an increased cellular G-actin content reduces FLNA phosphorylation at S2152 and thereby favors DLC1-FLNA complex formation.

### Enhanced FLNA phosphorylation at S2152 in tumor xenografts

The obtained results indicate that FLNA pSer^2152^ may be a valuable target for the therapy of DLC1-deficient cancers. To assess phosphorylation of FLNA at Ser2152 *in vivo*, we employed a subcutaneous xenograft mouse model. To validate the expression of DLC1 in the tumor versus liver tissue, we analyzed the expression of DLC1 by immunohistochemistry. DLC1 expression was detectable in liver tissue, but almost completely abolished in the tumors (Fig. 7A). Tumor sections negative for DLC1 exhibited increased FLNA pSer^2152^ levels (Fig. 7A). In contrast, liver control tissues exhibited reduced FLNA pSer^2152^ levels (Fig. 7A). Immunoblot analysis of tumor and liver tissue validated an inverse correlation between FLNA pSer^2152^ and DLC1 expression in tumor versus control tissues (Fig. 7B). Together, these results suggest that loss of DLC1 and phosphorylation of FLNA at Ser2152 are likely to be pro-tumorigenic events.

## Discussion

Our study identifies FLNA as a novel interaction partner of DLC1, which enhances its RhoGAP activity, thereby suppressing MRTF-A/SRF transcriptional activity and promoting the induction of cellular senescence. We show that FLNA phosphorylation at Ser^2152^ dissociates the DLC1-FLNA complex while favoring FLNA’s interaction with MRTF-A, thus facilitating SRF-driven transcription. In vivo, increased levels of phosphorylated FLNA correlated with reduced DLC1 expression, underscoring the clinical relevance of this posttranslational modification. This inverse relationship is consistent with our previous studies, in which low DLC1 expression in HCC tissues corresponded with enhanced nuclear localization of MRTF-A [40]. These findings provide deeper insight into the molecular mechanisms underlying DLC1 regulation and highlight FLNA pSer^2152^ as a valuable biomarker and promising therapeutic target in HCC.

Our domain-mapping experiments revealed that FLNA’s actin-binding domain (ABD) and DLC1’s RhoGAP domain are essential for their interaction. Both regions are critical for their localization at focal adhesions and regulation of cytoskeletal dynamics [9]. Consistent with this, the FLNA non-binding DLC1ΔRhoGAP mutant, unlike DLC1 WT, failed to suppress focal adhesion or stress fiber formation. DLC1’s tumor-suppressive effects are known to depend on its localization at focal adhesions and interaction with proteins such as talin and focal adhesion kinase (FAK) [31,46]. FLNA, a key modulator of focal adhesion remodeling, has been shown to influence RhoA activity through direct interactions [47]. Based on our findings, we propose that FLNA functions as a scaffold protein that stabilizes DLC1’s localization at focal adhesions. By binding to DLC1’s RhoGAP domain, FLNA may enhance the spatial proximity of DLC1 to RhoA, thereby promoting RhoGTP hydrolysis and suppression of RhoA-mediated signaling. In addition, recent studies have shown that DLC1’s RhoGAP domain interacts with p120RasGAP, thereby negatively regulating its ability to associate with RhoA [48]. Collectively, these findings highlight that beyond its functional role in catalyzing RhoGTP hydrolysis, the RhoGAP domain serves as a central binding site for protein interactions that modulate DLC1’s tumor suppressor activity.

Moreover, our data reveal that DLC1 suppresses MRTF-A/SRF signaling through FLNA binding. FLNA exerts a dual role in this pathway, functioning either as a suppressor or activator of MRTF-A/SRF signaling, depending on its binding partners and phosphorylation status. While FLNA binding to DLC1 inhibits SRF activity, its association with MRTF-A promotes SRF transcriptional activity [7]. Our data substantiates the notion of a bivalent role for FLNA, consistent with other reports that discuss its multifaceted involvement in cancer, where it has been implicated in both tumor-promoting and tumor-suppressive signaling [[49], [50], [51], [52]]. We identified FLNA phosphorylation at Ser^2152^ as the key mechanism orchestrating the interplay between the DLC1-FLNA and MRTF-A-FLNA complexes. This phosphorylation event determines whether FLNA suppresses or promotes MRTF-A/SRF signaling by associating with its interaction partners. Other studies displayed a similar role of FLNA phosphorylation in oncogenic signaling pathways. In pituitary tumors, FLNA pSer^2152^ modulates its interaction with SST2, subsequently suppressing its inhibitory signaling, resulting in increased cell migration and proliferation [12]. Therefore, we propose that FLNA phosphorylation could serve as a key mechanism orchestrating FLNA’s role in oncogenic signaling. These findings open up important therapeutic considerations for the regulation of FLNA phosphorylation. For instance, cyclosporin A (CsA), a well-characterized calcineurin inhibitor, prevents FLNA dephosphorylation [18], which may, in turn, promote HCC progression by activating MRTF-A/SRF transcription. Supporting this, CsA treatment has been shown to increase tumor angiogenesis, cell motility, and invasiveness, effects governed through transforming growth factor-beta (TGF-beta) production [53,54]. Since TGF-beta expression is transcriptionally regulated by the MRTF-A/SRF axis [6], it is tempting to speculate that CsA exerts its tumor-promoting effects by this pathway. In contrast, Protein Kinase B (Akt) induces FLNA pSer^2152^ and simultaneously attenuates DLC1 function [27,43]. Akt targets multiple residues (S^298^, S^329^, and S^567^) within the serine-rich region, modifications that impair DLC1’s RhoGAP activity [27]. Thus, targeting Akt could offer a dual therapeutic benefit by preventing FLNA phosphorylation and restoring DLC1 activity, suggesting that Akt inhibitors such as MK-2206 could represent promising therapeutic agents for HCC treatment.

To investigate whether DLC1 activity can be exploited therapeutically, we employed a DLC1 binding peptide, which was shown to restore DLC1 function [28]. Peptide induced DLC1 activation facilitated DLC1-FLNA complex formation, leading to actin filament disassembly. This was accompanied by MRTF-A-FLNA complex dissociation and suppression of MRTF-A/SRF transcriptional activity, resulting in reduced cell migration and senescence induction.

Our findings reveal that the balance between monomeric G-actin and polymerized F-actin controls FLNA phosphorylation at S2152 and thereby its binding preference towards DLC1 or MRTF-A. Treatment with the actin polymerization inhibitor Latrunculin B suppressed FLNA pSer^2152^ and induced DLC1-FLNA complex formation. In contrast, enhanced actin polymerization, achieved through expression of the F-actin stabilizing S14C actin mutant [44], reversed the effect on the FLNA pSer^2152^ status. Although the functional link of FLNA pSer^2152^ on FLNA’s actin-binding affinity remains to be fully elucidated, other studies suggest that it enhances FLNA’s interaction with F-actin [55].

Our data support a model illustrated in Fig. 7 in which actin polymerization drives FLNA phosphorylation at S2152 to promote F-actin binding, thereby displacing DLC1 from FLNA and attenuating its RhoGAP activity. Consistent with this, neuronal cell migration was found to rely on FLNA’s interaction with F-actin [55]. These results further expand the crucial role of actin dynamics in MRTF-A/SRF signaling, where F-actin polymerization drives MRTF-A nuclear localization and nuclear actin polymerization SRF transcriptional activity [[56], [57], [58]].

The observation that actin depolymerization increases DLC1-FLNA binding and and lowers FLNA pSer^2152^ levels raises the intriguing possibility that FLNA contributes to DLC1’s post-translational stability. In recent years, the therapeutic potential of restoring DLC1 expression by inhibiting its proteasomal degradation across various cancer types has gained increased attention [23,24]. Notably, the histone methyltransferase EZH2 was identified as part of a mechanism that promotes DLC1 destabilization through methylation at Lys^678^ (K678E) within the RhoGAP domain [23]. As FLNA has been previously implicated in the ubiquitin proteasome-mediated degradation of other proteins such as Ras-GFR1, a guanine-nucleotide exchange factor for Ras [59], future research is warranted to explore whether FLNA, particularly its phosphorylation status, regulates DLC1 stability.

One of the most intriguing aspect gleaned from this study is that elevated FLNA pSer^2152^ levels correlated with reduced DLC1 expression in mouse xenografts, which is consistent with a phosphoproteomic analysis linking FLNA phosphorylation to high metastasis and poor prognosis in patients [60]. This supports the model depicted in Fig. 7 in which actin polymerization drives FLNA phosphorylation at serine 2152, leading to DLC1-FLNA complex dissociation and enhanced MRTF-A/SRF signaling – a key driver of HCC progression. Thus, FLNA phosphorylation at S2152 could serve as a promising tumor biomarker and be exploited therapeutically in the future.

Our study opens up novel therapeutic avenues for targeting multiple nodes within the MRTF-A/SRF axis. Given the complex regulation of DLC1, which ranges from proteasomal degradation to posttranslational inactivation and actin dynamics, targeting both its expression and activation may be required for an effective therapeutic approach. Agents that prevent DLC1 degradation, such as proteasome inhibitors or actin-depolymerizing drugs, could be combined with DLC1 activators like the DLC1 binding peptides or inhibitors of Akt and Src [26]. These strategies aim to stabilize DLC1 and enhance its RhoGAP activity. They could be potentially paired with direct inhibitors of MRTF-A/SRF signaling. For instance, the TRPM7 channel, a known upstream inducer of this pathway, can be pharmacologically targeted using small-molecule inhibitors NS8593 and its improved structural derivatives, which we established as potent suppressors of SRF activity and HCC cell proliferation [6]. A combinatorial approach, including DLC1 stabilizing agents and MRTF-A/SRF inhibitors, offers a promising therapeutic strategy to suppress oncogenic signaling on multiple fronts. This approach could improve efficacy while potentially reducing toxicity and enabling a dose reduction of the therapeutic agents.

## Materials and methods

### Cell Culture, Transfections, and Reagents

HuH7 and HepG2 cells were maintained in RPMI 1640 medium (Sigma-Aldrich, Taufkirchen, Germany), while HuH6, HLF, HeLa, and Hep3B cells were cultured in Dulbecco’s Modified Eagle Medium (DMEM; Sigma-Aldrich, Taufkirchen, Germany). M2 and HEK293T cells were grown in Minimum Essential Medium Eagle (MEM; Sigma-Aldrich, Taufkirchen, Germany). All media were supplemented with 10% fetal bovine serum (FBS; Invitrogen, Karlsruhe, Germany) and 1% penicillin/streptomycin (Sigma-Aldrich, Taufkirchen, Germany). Transfections with siRNA and plasmids were carried out using Lipofectamine 2000 or Lipofectamine RNAiMAX (Invitrogen Karlsruhe, Germany) following the manufacturer’s instructions. The plasmids used are listed in the Supplementary information (Supplementary Table S1). The following reagents were used: Latrunculin B (LatB) (Merck KGaA, Darmstadt, Germany), epidermal growth factor (EGF), and lysophosphatidic acid (LPA) (Sigma-Aldrich, Taufkirchen, Germany).

### DNA cloning and plasmids

The GFP-DLC1 ΔRhoGAP (Δ639-789) construct was generated using the Q5® Site-Directed Mutagenesis Kit (New England Biolabs GmbH, Frankfurt am Main, Germany). GFP-DLC1 ΔRhoGAP was derived from the pEGFP-C1-DLC1 plasmid [55,56]. Primer sequences are listed in the Supplementary Information (Supplementary Table S2). The PCR product was cloned into pEGFP-C1-DLC1 by BamHI restriction. To generate GFP-DLC1-GAP (aa 625–876), the corresponding fragment was PCR-amplified from pEGFP-C1-DLC1 using primers described in the Supplementary Information (Supplementary Table S2). pGEX-FLNA-ABD construct was generated by subcloning from pCl-HA-FLNA-1-274 via EcoRI, NotI restriction sites, into the pGEX6P1 vector.

### Production of recombinant proteins

E. coli BL21 was transformed with pGEX vectors encoding GST-FLNA-ABD. Expression was induced with 0.1 mM IPTG overnight at room temperature (RT). Cells were harvested and lysed in PBS supplemented with Complete™ protease inhibitors (Roche, Mannheim, Germany) through sonication (three cycles, 10 s each, on ice). Following the addition of Triton X-100 (final concentration: 1%), lysates were clarified by centrifugation (8,000 × g, 10 min, 4°C). GST-tagged proteins were purified using glutathione sepharose resin (GE Healthcare, Düsseldorf, Germany) and eluted with 15 mM glutathione in 50 mM Tris-HCl (pH 8), followed by dialysis against 50 mM Tris-HCl. Protein concentrations were determined by measuring absorbance at 280 nm.

### F-/G-Actin fractionation assay

Cells were incubated with Actin lysis buffer (containing 50 mM MES, pH 6.8, 50 mM KCl, 1 mM EGTA, 1 mM MgCl_2_, 0.5% Triton X-100, and protease inhibitors (Merck KGaA, Darmstadt, Germany) with gentle shaking to solubilize the G-actin fraction. After incubation, the cells were harvested in Actin lysis buffer and sonicated to obtain the F-Actin fraction. Both fractions were analyzed by immunoblotting using an anti-actin antibody (Sigma-Aldrich, Merck, Darmstadt, Germany).

### RNA extraction, cDNA synthesis, and quantitative real-time PCR analysis

Total RNA was isolated using TRIzol Reagent (Invitrogen, Karlsruhe, Germany) according to the manufacturer’s instructions. 1 µg was reverse-transcribed into cDNA with SuperScript™ II Reverse Transcriptase (Invitrogen, Karlsruhe, Germany). Quantitative real-time PCR (qRT-PCR) was performed using a LightCycler® 96 System (Roche, Mannheim, Germany). Target gene expression levels were normalized to the 18S rRNA housekeeping gene. Gene-specific primers (Sigma-Aldrich, Taufkirchen, Germany) used for amplification are listed in the Supplementary Information (Supplementary Table S3).

### Peptide synthesis

Peptides were synthesized as C-terminal amides by Fmoc/tBu-based solid-phase synthesis, on TentaGel S RAM resin, using an automated multiple peptide synthesizer (ResPep by Intavis Inc.), as previously described [61].

### Immunoblotting

Proteins were denatured in Laemmli buffer at 95°C and separated by SDS-PAGE for 2 hours. Following electrophoresis, proteins were transferred onto polyvinylidene fluoride (PVDF) membranes (Merck KGaA, Darmstadt, Germany). To prevent non-specific binding, membranes were blocked with 5% non-fat milk in TBS-T and incubated with primary antibodies overnight at 4°C, followed by appropriate HRP-conjugated secondary antibodies (Supplementary Tables S4-S5). Chemiluminescent signals were detected using the ChemiDoc™ Imaging System (Bio-Rad, Feldkirchen, Germany).

### Senescence-associated ß-galactosidase staining

Cellular senescence was assessed 5 days after treatment using a β-galactosidase staining kit (Cell Signaling Technology, Danvers, MA, USA) according to the manufacturer’s protocol. Following staining, senescent cells exhibiting blue staining were identified under a bright-field microscope. The percentage of senescent cells was determined by counting 100 randomly selected cells.

### In situ proximity ligation assay (PLA)

Protein-protein interactions were detected using the DuoLink® In Situ Red Starter Kit Mouse/Rabbit (Sigma-Aldrich, Merck, Darmstadt, Germany) following the manufacturer’s instructions. Primary antibodies included anti-DLC1 (Santa Cruz Biotechnology, Santa Cruz, CA, USA), anti-FLNA (Thermo Fisher Scientific, Schwerte, Germany), and anti-MRTF-A (Santa Cruz Biotechnology, Santa Cruz, CA, USA). Samples were imaged using a fluorescence microscope (Nikon, Düsseldorf, Germany). PLA signals were quantified using ImageJ.

### Immunofluorescence (IF)

Cells were fixed with 4% paraformaldehyde in PBS for 10 minutes at room temperature (RT), followed by permeabilization with 0.1% Triton X-100 in PBS for 7 minutes. After blocking with 1% bovine serum albumin (BSA) in PBS for 30 min at 37°C, cells were incubated with primary antibodies (Supplementary Table S6) for 1 h at RT and appropriate fluorophore-conjugated secondary antibodies (Supplementary Table S7) for 45 min in the dark. F-actin filaments were stained with phalloidin coupled to Alexa Fluor 555 (Invitrogen, Karlsruhe, Germany), focal adhesions with anti-paxillin antibody (BD Biosciences, Heidelberg, Germany), and nuclei with 4’,6-diamidino 2-phenylindole (DAPI; Sigma-Aldrich, Taufkirchen, Germany). Coverslips were imaged using a confocal fluorescence microscope (Olympus, Hamburg, Germany). To quantify the ratio of nuclear-to-cytoplasmic MRTF-A, fluorescence intensities from nuclei and cytoplasm from 40 randomly selected cells were measured using ImageJ. Ratios were calculated by dividing the average nuclear intensity by cytoplasmic intensity.

### RhoA biosensor analysis

For analyzing RhoA activity, HeLa cells stably expressing the tetracycline (Tet)-inducible RhoA biosensor dimericTomato-2xrGBD were used. Cells were transfected with the indicated constructs for 48 hours, followed by biosensor induction through overnight doxycycline treatment. After fixation and staining, fluorescence intensities were analyzed using confocal microscopy (Olympus, Hamburg, Germany) and measured using ImageJ.

### FRET acceptor photobleaching fluorescence resonance energy transfer (FRET)

48 h after transient transfection, the cells were serum-starved for 18 h in media supplemented with 0.2% FBS. The cells were then treated with 20 µM LPA or H2O (negative control) for 45 min at 37°C. Following treatment, cells were fixed with 4% paraformaldehyde in Hanks' Balanced Salt Solution (HBSS) for 30 minutes at room temperature. Optical imaging was performed in HBSS at RT. The FRET acceptor dsRed was excited with a 514 nm Argon laser, and its emission was measured between 525-650 nm. ECFP was excited with a 405 nm laser, and its emission was detected between 450-490 nm. A Leica TCS SP5II AOBS confocal microscope and FRET AB-Wizard were used for sequential acquisition of images. Photobleaching was achieved by illuminating the cells ten times at 514 nm. FRET efficiency was calculated using a formula that considers the ECFP levels before and after photobleaching.

$$\text{FRETefficiency}=\frac{1-ECFPpre}{ECFPpost}$$

### Co-Immunoprecipitation (Co-IP)

Immunoprecipitation was performed using magnetic Dynabeads (Invitrogen, Thermo Fisher Scientific, Waltham, MA, USA). Dynabeads were washed with ice-cold PBS and incubated with the appropriate primary antibody (Supplementary Table S8) for 30 min with gentle rotation to facilitate antibody binding. At the same time, cells were harvested and lysed in 500 μL of immunoprecipitation buffer containing 0.2% phenylmethylsulfonyl fluoride, 150 mM NaCl, 50 mM Tris-HCl, a protease inhibitor cocktail (Merck KGaA, Darmstadt, Germany), and 10% glycerol. Upon incubation on ice for 45 minutes, lysates were centrifuged at 12,700 × g for 15 min at 4°C. Supernatants were incubated with the antibody-coated beads overnight at 4°C under rotation. After binding, the beads were washed with immunoprecipitation buffer to remove non-specifically bound proteins. Immunoprecipitated protein complexes were analyzed by SDS-PAGE followed by immunoblotting.

### Pull-down assay

HEK293T cells were transfected with GFP, GFP-DLC1 full-length, or GFP-DLC1-GAP plasmids. Whole-cell extracts were obtained by lysing cells in 1% NP-40 extraction buffer (NEB) (50 mM Tris-HCl, pH 7.5, 150 mM NaCl, 1% NP-40, 1 mM sodium orthovanadate, 10 mM sodium fluoride, and 20 mM β-glycerophosphate plus Complete™ protease inhibitors). Lysates were clarified by centrifugation at 13,000 g for 10 minutes. For pull-downs, whole-cell extracts were diluted with extraction buffer to a final concentration of 0.5% NP-40 and incubated with immobilized GST proteins for 2 hours. Beads were washed three times with 0.5% NEB and eluted by boiling in SDS-PAGE loading buffer**.**

### RhoGAP activity assay

RhoGAP activity assay was performed using the RhoGAP Assay kit (Cat. #BK105, Cytoskeleton, Denver, CO, USA) according to the manufacturer’s instructions. Briefly, recombinant His-DLC1-GAP domain was incubated with recombinant GST-fusion proteins for 15 min at RT. The reaction was initiated by adding RhoA and GTP, followed by incubation for 1 h at 37°C. After the addition of CytoPhos reagent and signal development for 10 min at RT, absorbance at 650 nm was measured.

### Luciferase Assay

SRF transcriptional activity was evaluated using a dual-luciferase reporter assay (Promega, Madison, WI, USA). Cells were seeded in 24-well plates and transfected with 0.125 µg of the 5xSRE Firefly luciferase reporter plasmid and 0.075 µg of the Renilla luciferase reporter plasmid driven by the simian virus 40 (SV40) promoter (internal control for transfection efficiency). After 24 h, cells were treated as indicated, and Renilla and Firefly luciferase activities were measured using the Dual-Luciferase Reporter Assay System (Promega, Madison, WI, USA) on the BioFix Lumi-10 luminometer (Macherey-Nagel, Düren, Germany). Firefly luciferase activity was normalized to the corresponding Renilla luciferase activity.

### Scratch Wound Assay

Cell migration was evaluated using a scratch wound assay. Cell monolayers in 6-well plates were scratched with a 200 µL pipette tip to create a linear wound. Detached cells were removed by gently washing the wells with PBS, followed by the addition of fresh medium containing the indicated treatments. Images of the wound area were captured at 0 and 24 or 48 hours post-scratch. Wound closure was quantified by measuring the scratch area at each time point with ImageJ software. The percentage of closure was calculated as follows:

$$\%\;\text{wound}\;\text{closure}=\frac{wound\;area\;\left(0\;h\right)-wound\;area\;\left(48\;h\right)}{wound\;area\;\left(0\;h\right)}\;x\;100$$

### Organoid culture

Human gastrointestinal biopsies were washed with cold PBS and incubated for 30 min at 4°C with 2 mM EDTA in PBS. The supernatant was then replaced with fresh PBS, centrifuged at 200 × g for 5 min at 4°C, and resuspended in Matrigel (Corning, New York, NY, USA). Afterwards, the organoids were cultured at 37°C in a 5% CO2 atmosphere with 250 µl medium (Organoid Growth Medium, Stem cell, Vancouver, Canada). The medium was replaced every 2–3 days, and organoids were passaged weekly. To generate monolayers for the PLA assay, the medium was aspirated and renewed with cell recovery solution (Corning, New York, NY, USA). The plate was then put on ice for 20 min in order to dissolve the Matrigel. Organoids were collected in cold PBS and centrifuged (200 × g for 5 min at 4°C). The pellet was washed, centrifuged, resuspended in TripLE Express (Thermo Scientific, Waltham, MA, USA), and incubated at 37°C for 15 min. Organoids were dispersed into single cells by pipetting. The single cells were centrifuged (200 × g, 5 min, 4°C), and the pellet was resuspended in organoid medium. The cells were seeded into precoated chamber slides (Matrigel, PBS 1:50; 1 h; 37°C; Thermo Scientific, Waltham, MA, USA).

### Mouse tumor xenografts

Tumor and non-tumorous liver control samples were lysed in RIPA buffer supplemented with freshly added protease inhibitors (Merck KGaA, Darmstadt, Germany) and sodium orthovanadate (Carl Roth GmbH, Karlsruhe, Germany) and homogenized using the TissueRuptor® (Qiagen, Hilden, Germany), followed by centrifugation and preparation of the supernatant for immunoblot analysis.

Animal experiments have been approved in advance by the ethics committee (CEEA– 067 Comité de Réflexion éthique en expérimentation animale, Approval # 26842), and the animals’ health status was monitored throughout the experiments by a health monitoring program according to Federation of European Laboratory Animal Science Associations (FELASA) guidelines. Animals were housed in 501 cm² ventilated cages under controlled environmental conditions (22 ± 2°C, 55 ± 10% humidity, 12/12 h light/dark cycle), with ad libitum access to food and water. Mice were euthanized by intraperitoneal injection of a pentobarbital overdose when tumor volume reached 2000 mm³, and tumors and livers were collected and preserved flash-frozen or in 4% paraformaldehyde (PFA).

### Immunohistochemistry

Formalin-fixed, paraffin-embedded tissue sections were deparaffinized in xylene and rehydrated through graded ethanol. Next, the sections were heated in Target Retrieval Solution (Dako, Santa Clara, CA, USA). Endogenous peroxidase activity was quenched with 3% hydrogen peroxide, and non-specific binding was blocked using FBS. Sections were incubated overnight at 4°C with primary antibodies (Supplementary Table S9), followed by incubation with the appropriate biotinylated secondary antibodies. Detection was carried out using the VECTASTAIN® Elite® ABC Kit and DAB substrate (Vector Laboratories, Burlingame, CA, USA). Slides were counterstained with hematoxylin, dehydrated in graded ethanol, cleared in xylene, and coverslipped using Entellan® (Merck KGaA, Darmstadt, Germany).

### Statistical analysis

Statistical analysis was performed using a two-sided Student’s t-test, one-way ANOVA followed by Tukey’s post-hoc test, or closed testing, as appropriate. Unless stated otherwise, data were analyzed from three independent experiments. Values are presented as mean and standard deviation (mean ± SD) or standard error of the mean (mean ± SEM) and were considered significant with *p<0.05, **p<0.01, and ***p<0.001.

## Data and materials availability

All data needed to evaluate the conclusions in the paper are present in the paper or the Supplementary Materials.

## CRediT authorship contribution statement

**Michael Sergeev:** Writing – review & editing, Visualization, Methodology, Investigation, Data curation. **Melanie A. Meier:** Visualization, Investigation, Formal analysis. **Petra Wohlleben:** Visualization, Methodology, Investigation, Formal analysis. **Laura Rupprecht:** Methodology, Investigation, Formal analysis. **Mirka Kupraszewicz-Hutzler:** Methodology, Investigation. **Karl Hilgers:** Supervision, Investigation. **Andrea Hartner:** Writing – review & editing, Visualization, Investigation. **Anna-Lena Voegele:** Methodology, Investigation. **Raja Atreya:** Supervision, Methodology. **Yannick Frey:** Methodology. **Showmika Srirangan:** Methodology, Investigation. **Jutta Eichler:** Supervision, Methodology. **Caroline Confais:** Methodology, Investigation. **Benoît Hédan:** Supervision, Methodology. **Ulrich Jarry:** Supervision, Methodology. **Monilola A. Olayioye:** Supervision, Methodology. **Susanne Muehlich:** Writing – review & editing, Writing – original draft, Visualization, Supervision, Project administration, Investigation, Formal analysis, Conceptualization.

## Declaration of competing interest

I, Susanne Muehlich, am aware of the conflict of interest policy and my obligations under it. I declare to the best of my knowledge the information I have provided is true and correct.

## Appendix Supplementary materials

Image, application 1
