# Supplementary material for: Filamin a binds deleted in liver cancer 1 (DLC1) to promote its tumor suppressor activity and inhibit the SRF coactivator MRTF-A
Source: Neoplasia. 2025 Nov 28;71:101258. doi: 10.1016/j.neo.2025.101258 (PMC12704088; doi:10.1016/j.neo.2025.101258)
Supplement: Supplementary file 1 [file mmc1.docx]

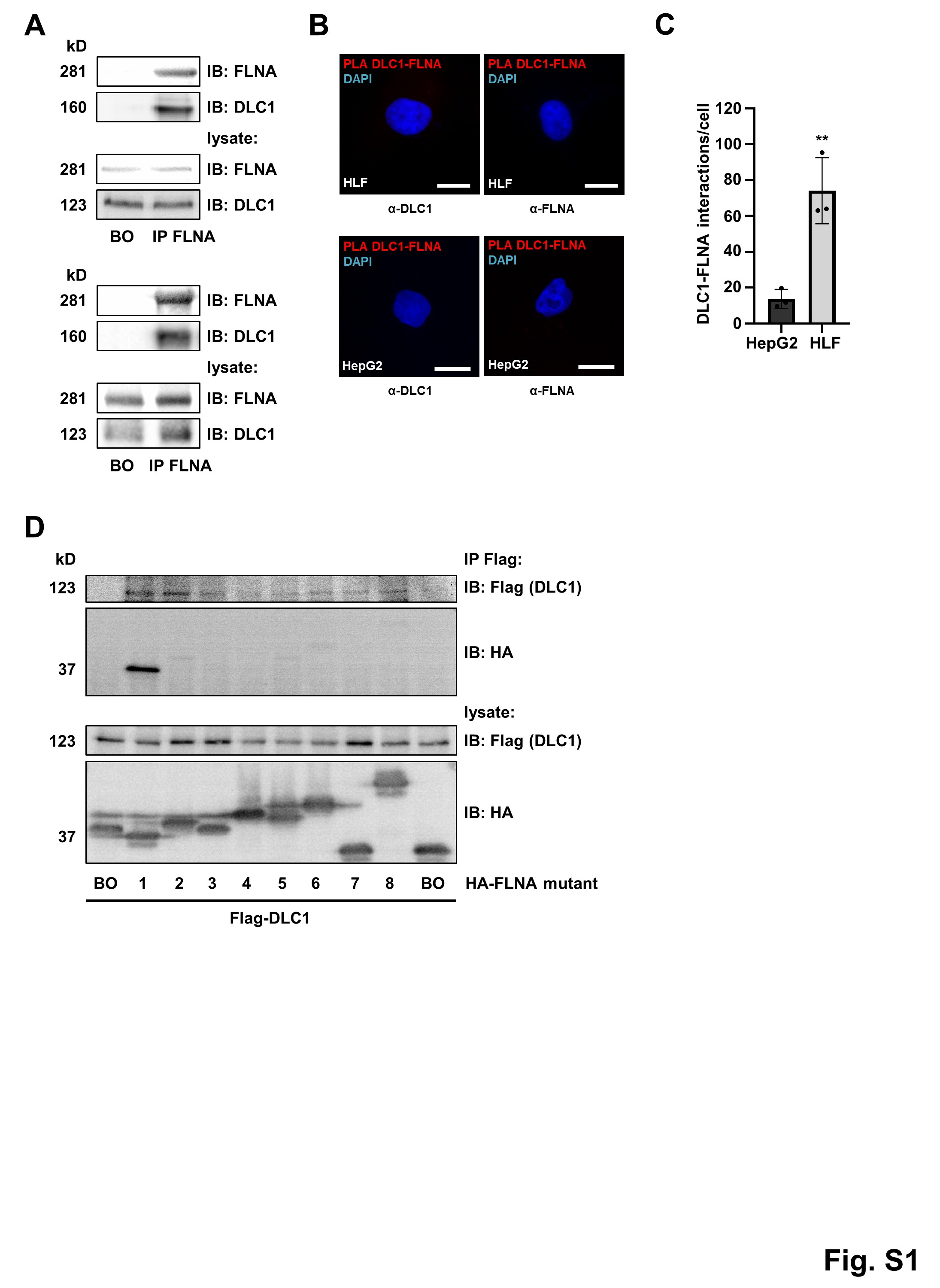


**Supplementary Figure S1:** Endogenous DLC1-FLNA complex and mapping of the FLNA binding sites required for DLC1 association. (A) Detection of endogenous DLC1-FLNA interaction in HLF (top) and HuH7 (bottom) cells. Co-immunoprecipitation (IP) for FLNA followed by immunoblot (IB) analysis using anti-DLC1 and anti-FLNA antibodies. BO: Sepharose beads only (no antibody). (B) Control proximity ligation assays (PLA) for endogenous DLC1-FLNA interaction in HepG2 and HLF cells, using only anti-DLC1 or anti-FLNA antibodies, respectively. DAPI was used for nuclear staining. Scale bar: 10 µm. (C) Quantification of PLA signals for endogenous DLC1-FLNA interactions shown in Fig. 1B. Values are mean ± SD (n=3); **p<0.01. (D) Mapping of the FLNA binding sites required for DLC1 binding. IP using anti-Flag antibody in HuH7 cells co-transfected with Flag-DLC1 and different HA-FLNA fragments 1-8. 1: FLNA a.a. 2-275; 2: FLNA a.a. 276-570; 3: FLNA a.a. 571-866; 4: FLNA a.a. 867-1154; 5: FLNA a.a. 1155-1442; 6: FLNA a.a. 1443-1778. 7: FLNA a.a. 1779-2284; 8: FLNA a.a. 2285-2751. Immunoblotting was performed using anti-Flag and anti-HA antibodies. BO: Dynabeads-only control (no antibody).


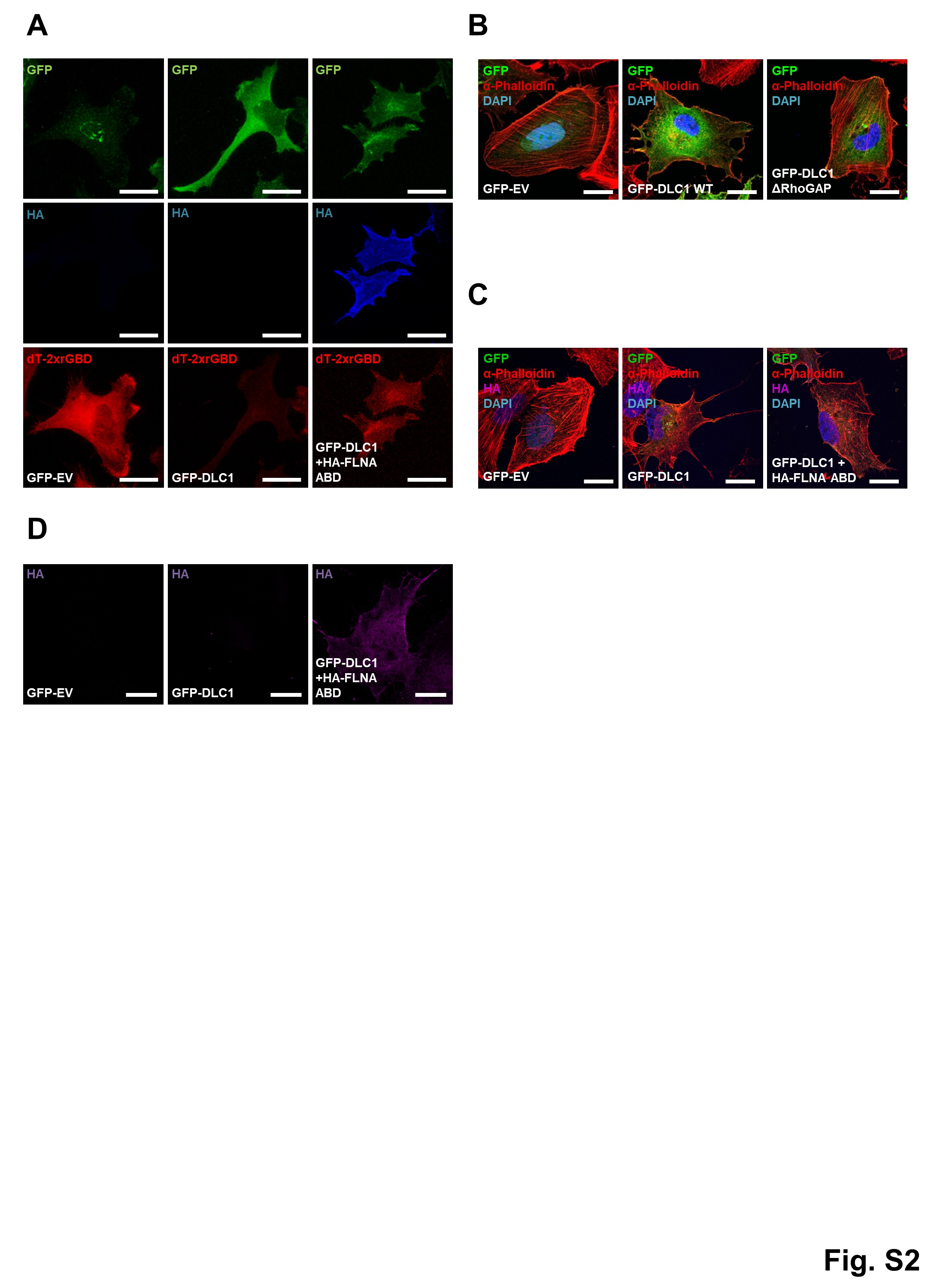


**Supplementary Figure S2:** DLC1-FLNA complex suppresses RhoA activation and stress fiber formation. (A) Representative images of Fig. 2B. HeLa cells stably expressing the Rho biosensor dT-2xrGBD under a tetracycline-response element (TRE) were transfected with GFP-DLC1 and HA-FLNA-ABD or empty vector (GFP-EV), followed by treatment with 1 µg/ml doxycycline for 18 hours to induce dT-2xrGBD expression. Immunofluorescence analysis was performed using an anti-HA antibody. Scale bar: 20 µm. (B) Phalloidin staining in HuH7 cells transfected with GFP-DLC1 WT, GFP-DLC1ΔRhoGAP, or GFP-empty vector (GFP-EV). DAPI was used for nuclear staining. Scale bar: 20 µm. (C) Phalloidin staining in HuH7 cells transfected with GFP-empty vector (GFP-EV) or GFP-DLC1 WT with HA-FLNA ABD, or -empty vector. DAPI was used for nuclear staining. Scale bar: 20 µm. (D) Representative images of HA staining from HuH7 cells expressing GFP DLC1 WT together with HA-FLNA ABD or –empty vector (EV), corresponding to the focal adhesion staining shown in Fig. 2D.


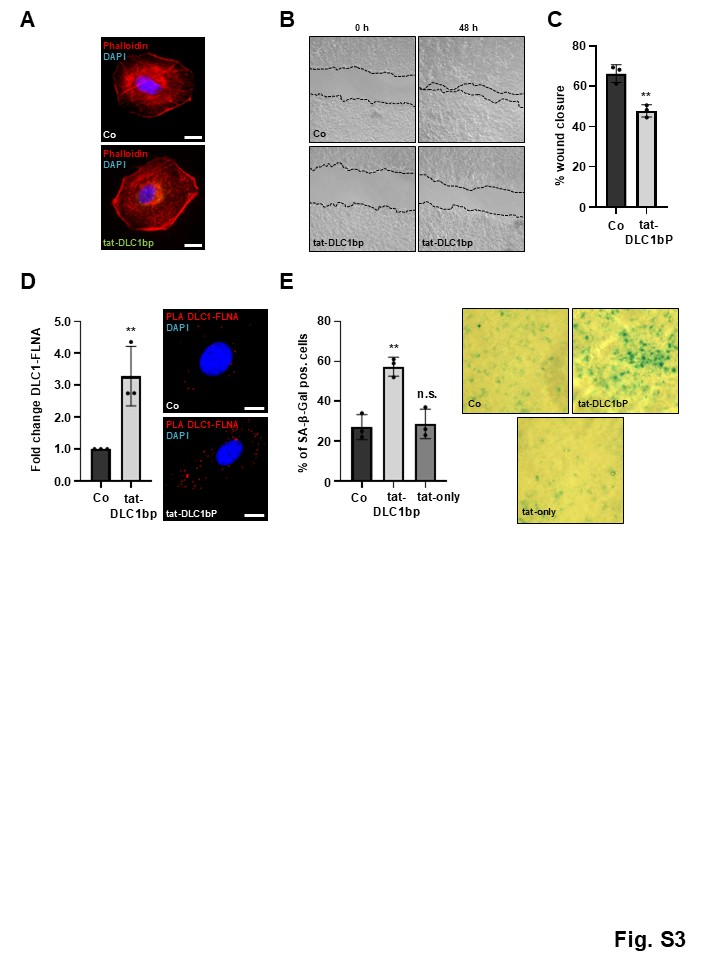
**Supplementary Figure S3:** DLC1 activation via DLC1-binding peptides promotes DLC1-FLNA association. (A) Phalloidin staining of F-actin in Hep3B cells treated with 20µM DLC1 binding peptide (tat-DLC1bp) or DMSO (Co). DAPI was used for nuclear staining. Scale bar: 20 µm. (B) Representative images for Fig. 3C. HuH7 cells treated with 20 µM tat-DLC1bp or DMSO (Co) were subjected to scratch-wound assay. Images were taken at 0 and 48 h post-scratch. Quantification see Fig. 3C. (C) Quantification of wound closure in HuH6 cells treated with 20µM tat-DLC1bp or DMSO (Co). Values are mean ± SD (n=3); **p<0.01; Student’s t-test was performed for statistical analysis. (D) Quantification of proximity ligation assay (PLA) for endogenous DLC1 and FLNA in HuH7 cells treated with 20µM tat-DLC1bp for 4 h or DMSO (Co). Representative PLA images (right). DAPI was used for nuclear staining. Scale bar: 20µm. Values are mean ± SD (n=3); **p<0.01; Student’s t-test was performed for statistical analysis. (E) Quantification of senescence-associated β-galactosidase staining in HuH7 cells treated with 30 µM tat-DLC1bp, tat control peptide (tat-only), or DMSO (Co) every 48h for 5 days (left). Representative images of SA-β-gal staining (right). The number of SA-β-gal-positive blue cells was counted. Values are mean ± SD (n=3); **p<0.01; One-way ANOVA followed by Tukey’s post-hoc test was used for statistical analysis.


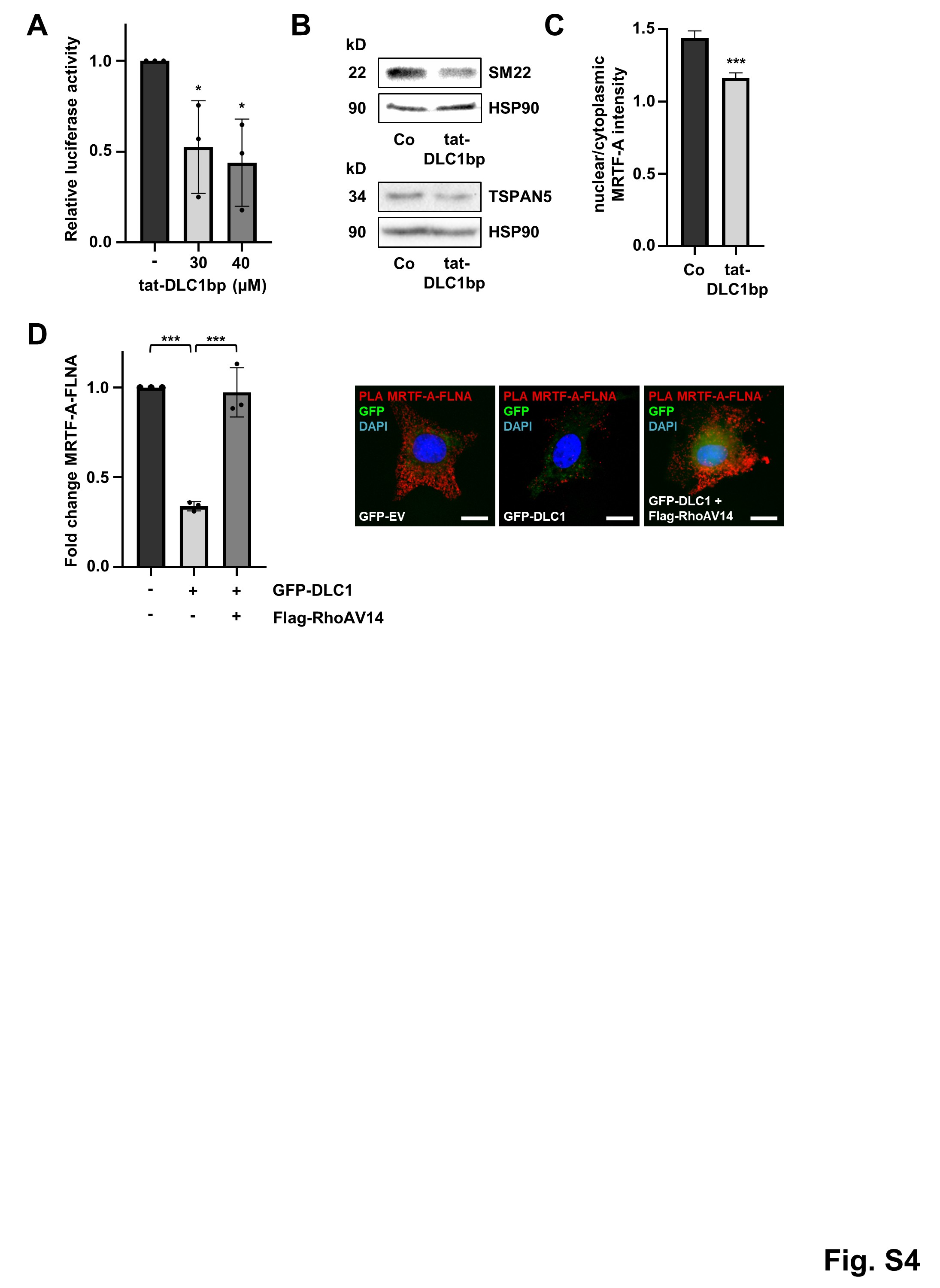


**Supplementary Figure S4:** DLC1-FLNA complex formation inhibits MRTF-A/SRF transcriptional activity. (A) Quantification of luciferase activity in HuH6 cells co-expressing an SRE-dependent luciferase reporter gene (5xSRE) and a Renilla luciferase internal control (pRL-SV40P) following 24 h treatment with different concentrations of DLC1 binding peptide (tat-DLC1bp). Firefly luciferase activity was normalized to Renilla luciferase activity. Values are mean ± SD (n = 3); *p<0.05; Student’s t-test was performed for statistical analysis. (B) Immunoblot analysis for TSPAN5, SM22, and HSP90 in lysates of HuH6 cells treated with 30µM tat-DLC1bp or DMSO (Co) for 24 h. (C) Quantification of nuclear-to-cytoplasmic intensity of MRTF-A in HuH7 cells treated with 30 µM tat-DLC1bp or DMSO (Co) for 24 h. Values are mean ± SEM (n=40); ***p<0.001; Student’s t-test was performed for statistical analysis. (D) Proximity ligation assay (PLA) for MRTF-A-FLNA interactions in HuH7 cells transfected with GFP-empty vector or GFP-DLC1, with or without constitutively active Flag-RhoAV14. DAPI was used for nuclear staining. Scale bar: 20 µm. Values are mean ± SD (n=3); **p<0.01, ***p<0.001; One-way ANOVA followed by Tukey’s post-hoc test was used for statistical analysis.

**
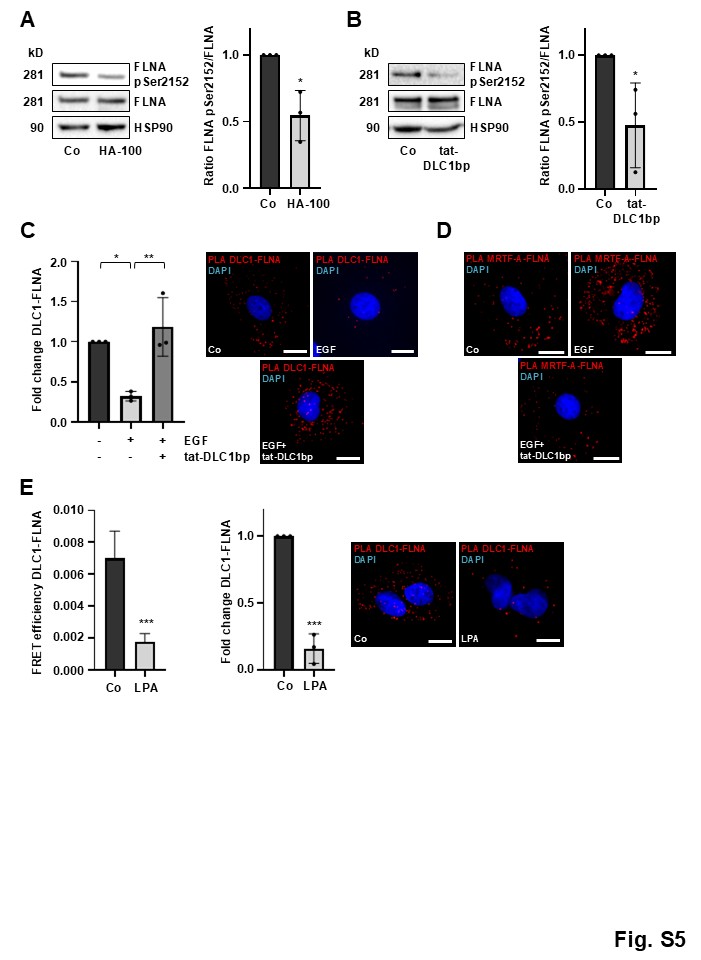
Supplementary Figure S5:** FLNA phosphorylation governs the equilibrium between the DLC1-FLNA and MRTF-A-FLNA complexes. (A) Lysates from HuH7 cells treated with 10 µM HA-100 or DMSO (Co) were immunoblotted using anti-FLNA pSer2152, anti-FLNA, and anti-HSP90 antibodies. The FLNApSer2152/FLNA ratio was quantified and normalized to HSP90. Values are mean ± SD (n = 3); *p<0.05; Student’s t-test was performed for statistical analysis. (B) Immunoblot analysis for FLNA pSer2152, total FLNA, and HSP90 in HuH6 cells treated with 30 µM tat-DLC1-binding peptide (tat-DLC1bp), or DMSO (Co) for 24 h. The FLNApSer2152/FLNA ratio was quantified and normalized to HSP90. Values are mean ± SD (n = 3); *p<0.05; Student’s t-test was performed for statistical analysis. (C) Analysis of proximity ligation assay (PLA) for endogenous DLC1-FLNA signals in Hep3B cells treated as described in Fig. 5D. DAPI was used for nuclear staining. Scale bar: 20 µm. Values are mean ± SD (n = 3); *p<0.05, ***p<0.001; One-way ANOVA followed by Tukey’s post-hoc test was used for statistical analysis. (D) Representative images from endogenous MRTF-A-FLNA interactions analyzed in Fig. 5E. DAPI was used for nuclear staining. Scale bar: 20 µm. (E) Serum-starved HepG2 cells expressing Myc-FLNA and Flag-DLC1 were stimulated with 20µM LPA for 45 min, and FRET efficiency was measured (left). Values are mean ± SEM (n = 3); ***p < 0.001. PLA analysis for endogenous DLC1 and FLNA for serum-starved HepG2 cells after stimulation with 20µM LPA for 45 min (right). DAPI was used for nuclear staining. Scale bar: 20 µm. Values are mean ± SD (n = 3); ***p < 0.001; Student’s t-test was performed for statistical analysis.

**Supplementary Tables**

**Table S1:** DNA plasmids

| **plasmids** | **Provided** | **Published** |
| --- | --- | --- |
| Flag-DLC1 | Previously described | Scholz et al, J. Cell Sci. 2009 |
| Flag-R62D-actin | Gift from Guido Posern | Posern et al, Mol Biol Cell. 2002 |
| Flag-RhoAV14 | Gift from Ron Prywes | - |
| Flag-S14C-actin | Gift from Guido Posern | Posern et al, Mol Biol Cell. 2002 |
| GFP-DLC1 ΔRhoGAP | described in material and methods | unpublished |
| GFP-DLC1 K714E | Previously described | Holeiter et al, Cancer Res. 2008 |
| GFP-DLC1 WT | Previously described | Holeiter et al, Cancer Res. 2008 |
| GFP-EV | Clontech Laboratories, Mountain View, California, USA | - |
| HA-FLNA mutants 1-8 | Gift from Fred Berry | Kircher et al., Sci Signal. 2015 |
| mCherry-FLNA d571-866 | Previously described | Konopa et al., Oncogenesis 2022 |
| mCherry-FLNA WT | Gift from Michael Davidson (Addgene plasmid #55047; http://n2t.net/addgene:55047; RRID: Addgene_55047) | Konopa et al., Oncogenesis 2022 |
| Myc-FLNA | Gift from John Blenis (Addgene plasmid #8982; http://n2t.net/addgene:8982; RRID: Addgene_8982) | Woo et al, Mol Cell Biol. 2004 |
| Myc-FLNA S2152A | Gift from John Blenis (Addgene plasmid #8983; http://n2t.net/addgene:8983; RRID: Addgene_8983) | Woo et al, Mol Cell Biol. 2004 |

**Table S2:** Primer sequences used for DNA cloning

| **primer** | **sequence** |
| --- | --- |
| GFP-DLC1 ΔRhoGAP Fw  GFP-DLC1 ΔRhoGAP Rv | 5’-CTC AAC ACC CTG AAG AGA G-3’  5’-CAC ACT CCG GTC CTT GTA-3’ |
| GFP-DLC1 GAP Fw  GFP-DLC1 GAP Rv | 5′ CGCGGATCCAAGAGGATCAAGGTTCCAGAC-3′ 5′ CGCGGATCCTCACAGGTGCCCGAGTGCTTC-3′ |

**Table S3:** Primer sequences used for qRT-PCR

| **primer** | **sequence** |
| --- | --- |
| 18S Fw  18S Rv | 5’-TCG AGG CCC TGT AAT TGG AAT-3’  5’-CCC TCC AAT GGA TCC TCG TTA-3’ |
| CXCL10 Fw  CXCL10 Rv | 5’-CCC CAC GTT TTC TGA GAC AT-3’  5’-TGG CAG TTT GAT TCA TGG TG-3’ |
| TNFSF10 Fw  TNFSF10 Rv | 5’-TTC ACA GTG CTC CTG CAG TC-3’  5’-GCC ACT TTT GGA GTA CTT GTC C-3’ |

**Table S4:** Primary antibodies used for immunoblotting

| **antibody** | **manufacturer** |
| --- | --- |
| anti-Actin (rabbit) | Merck, Darmstadt, Germany |
| anti-DLC1 (mouse) | BD Biosciences, Heidelberg, Germany |
| anti-Flag (mouse) | Sigma Aldrich, Taufkirchen, Germany |
| anti-FLNA (mouse) | Merck, Darmstadt, Germany |
| anti-FLNA pSer2152 (rabbit) | Cell Signaling Technology, Danvers, MA, USA |
| anti-GFP (rabbit) | Abcam, Cambridge, UK |
| anti-HA (mouse) | Santa Cruz Biotechnology, Santa Cruz, CA, USA |
| anti-HSP90 (mouse) | Santa Cruz Biotechnology, Santa Cruz, CA, USA |
| anti-mCherry (rabbit) | Abcam, Cambridge, UK |
| anti-Myc (mouse) | Cell Signaling Technology, Danvers, MA, USA |
| anti-SM22/Transgelin (rabbit) | GeneTex, Irvine, CA, USA |
| anti-TSPAN5 (rabbit) | Invitrogen, Karlsruhe, Germany |

**Table S5:** Secondary antibodies used for immunoblotting

| **antibody** | **manufacturer** |
| --- | --- |
| anti-mouse IgG, HRP-linked antibody | Cell Signaling Technology, Danvers, MA, USA |
| anti-rabbit IgG, HRP-linked antibody | Cell Signaling Technology, Danvers, MA, USA |

**Table S6:** Primary antibodies used for immunofluorescence and proximity ligation assay

| **antibody** | **manufacturer** |
| --- | --- |
| anti-DLC1 (mouse) | Santa Cruz Biotechnology, Santa Cruz, CA, USA |
| anti-FLNA (mouse) | Merck KGaA, Darmstadt, Germany |
| anti-FLNA (rabbit) | Invitrogen, Karlsruhe, Germany |
| anti-HA (mouse) | Santa Cruz Biotechnology, Santa Cruz, CA, USA |
| anti-MRTF-A (mouse) | Santa Cruz Biotechnology, Santa Cruz, CA, USA |
| anti-Paxillin (mouse) | BD Biosciences, Heidelberg, Germany |
| anti-Phalloidin Alexa-555 conjugated | Invitrogen, Karlsruhe, Germany |

**Table S7:** Secondary antibodies used for immunofluorescence

| **antibody** | **manufacturer** |
| --- | --- |
| Donkey anti-mouse IgG, Alexa-488-linked antibody | Invitrogen, Karlsruhe, Germany |
| Donkey anti-mouse IgG, Alexa-555-linked antibody | Invitrogen, Karlsruhe, Germany |
| Donkey anti-mouse IgG, Alexa-647-linked antibody | Invitrogen, Karlsruhe, Germany |

**Table S8:** Primary antibodies used for pulldown in immunoprecipitation assays

| **antibody** | **manufacturer** |
| --- | --- |
| anti-Flag (mouse) | Sigma-Aldrich, Taufkirchen, Germany |
| anti-FLNA (mouse) | Merck KGaA, Darmstadt, Germany |
| anti-HA (mouse) | Santa Cruz Biotechnology, Santa Cruz, CA, USA |
| anti-mCherry (rabbit) | Abcam, Cambridge, UK |
| anti-Myc (mouse) | Cell Signaling Technology, Danvers, MA, USA |

**Table S9:** Primary antibodies used for immunohistochemistry and immunoblotting of mouse tumor xenografts and non-tumorous samples

| **antibody** | **manufacturer** |
| --- | --- |
| anti-DLC1 (mouse) | Atlas Antibodies, Stockholm, Sweden |
| anti-FLNA (rabbit) | Invitrogen, Karlsruhe, Germany |
| anti-FLNA pSer2152 (rabbit) | Invitrogen, Karlsruhe, Germany |
| anti-HSP90 (mouse) | Santa Cruz Biotechnology, Santa Cruz, CA, USA |

**Table S10**: Amino acid sequences of peptides

| **Peptide** | **Sequence** |
| --- | --- |
| tat-DLC1bp | Fluo^1^-Aoa^2^-S^3^YHKKYRSATRD-Aoa-YGRKKRRQRRRPP-NH_2_ |
| tat-only | Fluo-Aoa-YGRKKRRQRRRPP-NH_2_ |

^1^Fluo, fluorescein; ^2^Aoa, 8-amino-3,6-dioxa-octanoic acid; ^3^C244 of TNS3-C2 was replaced with serine to avoid oxidation.
